# Decreased Low-Density Lipoprotein Cholesterol Level Indicates Poor Prognosis of Severe and Critical COVID-19 Patients: A Retrospective, Single-Center Study

**DOI:** 10.3389/fmed.2021.585851

**Published:** 2021-05-26

**Authors:** Mengmeng Zhao, Zhen Luo, Hua He, Bo Shen, Jinjun Liang, Jishou Zhang, Jing Ye, Yao Xu, Zhen Wang, Di Ye, Menglong Wang, Jun Wan

**Affiliations:** ^1^Department of Cardiology, Renmin Hospital of Wuhan University, Wuhan, China; ^2^Cardiovascular Research Institute, Wuhan University, Wuhan, China; ^3^Hubei Key Laboratory of Cardiology, Wuhan, China; ^4^Department of Medical Affairs, Renmin Hospital of Wuhan University, Wuhan, China

**Keywords:** coronavirus disease 2019, low-density lipoprotein, dyslipidemia, cardiac injury, severe and critical COVID-19 patients, prognosis

## Abstract

Coronavirus disease 2019 (COVID-19) has become a global public health crisis. Reduced low-density lipoprotein cholesterol (LDL-C) levels were observed in COVID-19 patients. The present study aimed to explore the relationship between LDL-C levels and the prognosis of severe and critical COVID-19 patients. A total of 211 severe and critical COVID-19 patients were enrolled and divided into four groups according to the LDL-C levels, including 53 patients in Group A (LDL-C ≥ 2.71 mmol/L), 53 patients in Group B (2.28 ≤ LDL-C < 2.71 mmol/L), 53 patients in Group C (1.83 ≤ LDL-C < 2.28 mmol/L) and 52 patients in Group D (LDL-C < 1.83 mmol/L). LDL-C levels were lower in critically ill patients than in severe patients. The main symptoms before admission, characteristics on admission and comorbidities of enrolled patients did not differ among the four groups. Compared with patients with high LDL-C levels, patients with low LDL-C levels were more likely to have immune and inflammation dysfunction, renal dysfunction, liver dysfunction and cardiac dysfunction on admission. The proportions of patients with shock and acute cardiac injury, of those admitted to intensive care unit (ICU) and of those treated with mechanical ventilation were inversely related to LDL-C level. The mortality of COVID-19 patients increased with LDL-C reduction. Serum LDL-C levels of COVID-19 patients was negatively correlated with CRP level, but positively correlated with lymphocyte count, as shown by Pearson correlation analysis. Proportional hazard models showed that low LDL-C levels were associated with increased risk of hospitalization death, cardiac injury and admission to the ICU. Taken together, these results suggest that decreased LDL-C levels indicate poor prognosis of severe and critical COVID-19 patients.

## Introduction

Coronavirus disease 2019 (COVID-19) is caused by severe acute respiratory syndrome coronavirus 2 (SARS-CoV-2). It has become a global public health crisis (1). Globally, as of December 22, 2020, there were ~76 023 488 confirmed cases of COVID-19, including 1 694 128 deaths. The estimated mortality is ~2.2%, which is much lower than that of Middle East respiratory syndrome (MERS) in 2015 and severe acute respiratory syndrome (SARS) in 2003 (2, 3). Nevertheless, the total numbers of confirmed COVID-19 cases and deaths have been significantly larger than those of MERS and SARS (2, 3). Currently, the pandemic continues to spread in many countries. There are no effective drugs to treat or prevent COVID-19 (4). Identification of parameters signaling poor prognosis and then improving them has become a priority.

Many previous studies discussed the relationship between the prognosis of COVID-19 and metabolic-associated conditions such as hypertension and diabetes (5, 6). However, few reports focused on dyslipidemia in patients with COVID-19. It was reported that levels of total cholesterol (TC) in SARS patients decreased significantly compared with healthy individuals (7). Another study showed dyslipidemia in 25 recovered SARS patients 12 years after infection (8). However, this might have been associated with high-dose pulses of methylprednisolone (8). In addition, many studies have observed a decrease in TC, low-density lipoprotein cholesterol (LDL-C) and high-density lipoprotein (HDL-C) levels in COVID-19 patients (9–11). Moreover, in most studies, the higher the severity of COVID-19, the greater the reduction in LDL-C and/or HDL-C (9, 10, 12). LDL-C levels may predict poor outcomes of COVID-19 (9). In previous studies, patients were mostly grouped by severity of COVID-19 to observe the LDL-C level. However, there is a lack of reports on the clinical manifestations and prognosis of COVID-19 patients with different LDL-C levels. Therefore, in order to figure out the different prognosis of patients with different LDL-C level, we divided severe and critical COVID-19 patients into four groups based on LDL-C levels in this study. And we found that decreased LDL-C levels indicated poor prognosis and increased risk for cardiac injury in severe and critical COVID-19 patients.

## Materials and Methods

### Study Design and Participants

**Figure 1:**
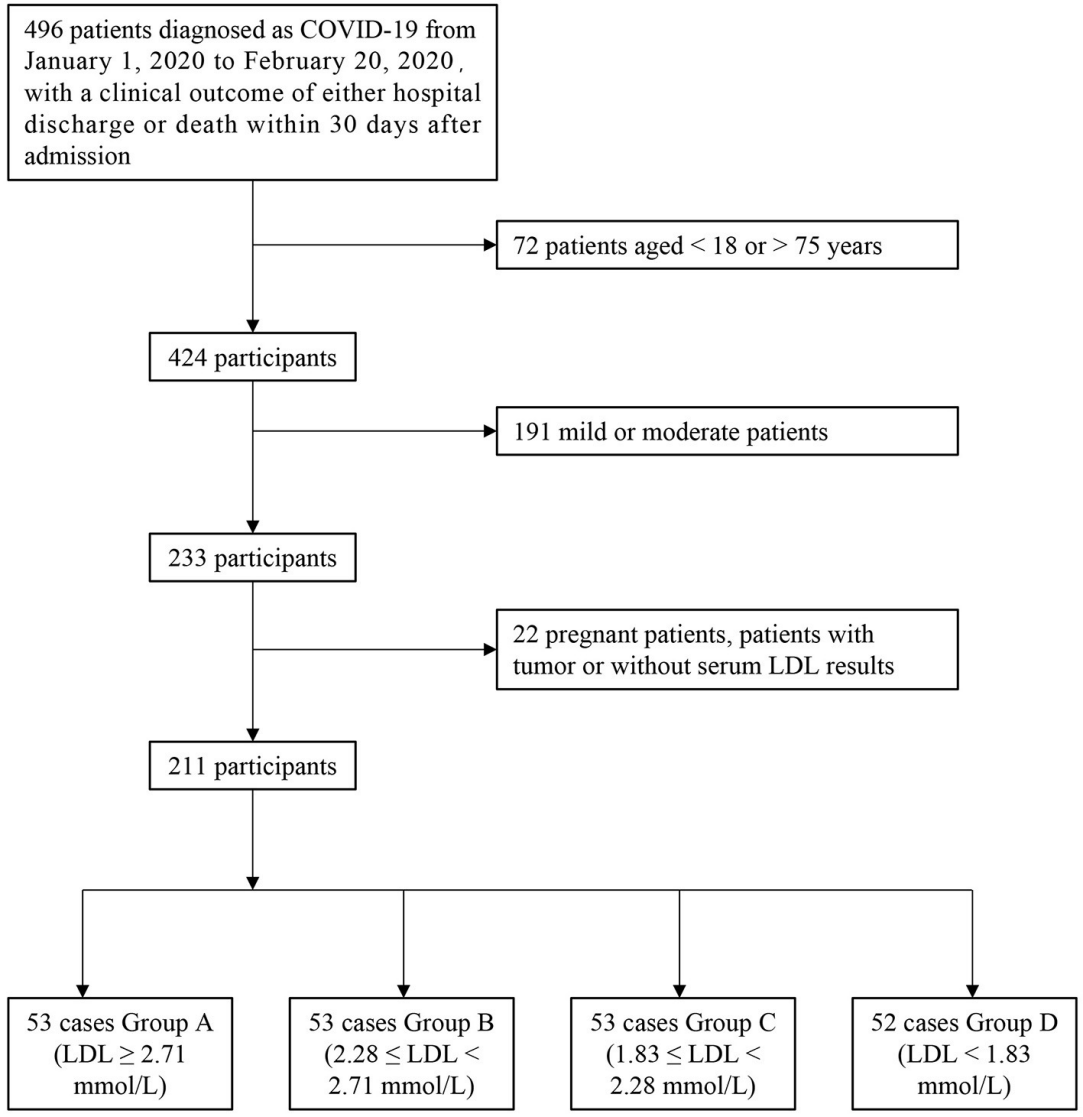
Study profile.

This retrospective study was approved by the Ethics Commission of Renmin Hospital of Wuhan University. We first included all confirmed COVID-19 patients admitted to Renmin Hospital of Wuhan University from January 1, 2020, to February 20, 2020, for whom a clinical outcome of either hospital discharge or death within 30 days after admission was recorded. Confirmed cases were those who had a positive result on a pathology test (real-time fluorescent RT-polymerase chain reaction detection of novel coronavirus nucleic acid or gene sequencing highly homologous with a known coronavirus) (13, 14). We then excluded case data that met the following criteria: (1) pregnant patients and patients with tumors; (2) patients younger than 18 years old or older than 75 years old; (3) patients without complete serum lipid data; and (4) mild and moderate patients. After applying various exclusion criteria, 285 patients were removed from the study. Of the remaining 211 patients, data were divided into four groups according to levels of LDL-C: Group A (LDL-C ≥ 2.71 mmol/L), Group B (2.28 ≤ LDL-C < 2.71 mmol/L), Group C (1.83 ≤ LDL-C < 2.28 mmol/L), and Group D (LDL-C < 1.83 mmol/L). The quartiles for LDL-C level were defined as the dividing lines (Figure 1).

### Criteria for Severity of COVID-19

According to the Diagnosis and Treatment Guidelines of Pneumonia Caused by Novel Coronavirus (6^th^ trial edition) (13), COVID-19 is generally classified into four types: mild, moderate, severe, and critical. Patients who met one of the following conditions were defined as severe: (1) dyspnea, breathing frequency 30 times per minute; or (2) finger oxygen saturation ≤ 93%. Patients who met any of the following conditions were defined as critical: (1) respiratory failure requiring mechanical ventilation; (2) convulsions; or (3) any concomitant organ failure other than respiratory failure, requiring monitoring and treatment in the intensive care unit (ICU). Other patients were classified as mild-to-moderate. In this study, we judged the severity according to the patient's condition throughout the course of illness but not the patient's condition upon admission.

### Criteria for Target Organ Injury

Diagnosis of shock was based on hypotension with failed volume resuscitation with administration of vasopressors to maintain blood pressure. High-sensitivity troponin I (hs-TnI) levels above 0.0796 ng/ml indicated acute cardiac injury (15). Alanine aminotransferase levels three-fold higher than normally indicated acute liver injury (ALI). Patients with one of the following conditions were diagnosed with acute kidney injury (AKI): (1) the highest serum creatinine (Scr) level increased by more than 26.5 μmol/L (0.3 mg/dl) within 48 h; (2) Scr exceeded the baseline value by 1.5-fold (confirmed or estimated to occur within 7 days); or (3) urine output <0.5 ml/kg ^*^ h, lasting more than 6 h.

### Data Collection

We obtained medical history, clinical symptoms, signs, laboratory tests, treatment, and outcome from the hospital's medical record system. The onset of COVID-19 was defined as the time when the associated symptoms first appeared. Outcomes or prognoses were collected within 30 days of admission. Laboratory tests were collected within 3 days after admission.

### Statistical Analysis

Continuous variables were expressed as medians and interquartile ranges (IQRs), and categorical variables were expressed as numbers (percentages). Continuous variables were analyzed using the Mann–Whitney *U* test. The classified variables were analyzed using the χ2 test or Fisher's exact test. A Pearson correlation analysis was used to calculate the correlation coefficient. Cox proportional hazard regression models were applied to determine the potential risk factors associated with the endpoints as shown by hazard ratio (HR) and 95% confidence interval (95% CI). The survive curves were conducted with the help of Graph Pad Prism. The statistical Software Package of Social Sciences (SPSS 26.0) was used for analysis, and a *p*-value <0.05 was considered statistically significant.

## Results

### Comparison of Basic Clinical Characteristics Among the Four Groups

A total of 211 severe and critically ill COVID-19 patients were enrolled in this study. We first calculated the quartiles for LDL-C levels [median (IQR), 2.28 (1.83, 2.71) mmol/L] and then divided all patients into four groups according to quartile, including 53 patients in Group A (LDL-C ≥ 2.71 mmol/L), 53 patients in Group B (2.28 ≤ LDL-C < 2.71 mmol/L), 53 patients in Group C (1.83 ≤ LDL-C < 2.28 mmol/L) and 52 patients in Group D (LDL-C < 1.83 mmol/L). There were 119 (56.4%) males and 92 (43.6%) females. The proportion of males was inversely proportional to LDL-C level (45.3 vs. 52.8 vs. 56.6 vs. 69.8%). The overall median (IQR) age was 63 [51, 69] years old. There were no differences among groups in terms of age. The median (IQR) intervals from disease onset to admission of the four groups were 13 (10,16), 10 (8,14), 10 (7,14), and 10 (7,12) days. Group D had a significantly shorter interval than did Group A (Table 1).

**Table 1:** Comparison of basic clinical characteristics among four groups.

**Parameters**	**All (*n* = 211)**	**A (*n* = 53)**	**B (*n* = 53)**	**C (*n* = 53)**	**D (*n* = 52)**
Male	119 (56.4%)	24 (45.3%)	28 (52.8%)	30 (56.6%)	37 (69.8%)^*^
Age, y	63 (51,69)	63 (52,67)	65 (56,70)	57 (47,67)	65 (55,69)
**Characteristics on admission**					
Temperature, °C^a^	36.7 (36.5,37.5)	36.7 (36.5,37.1)	36.7 (36.4,37)	36.9 (36.4,37.7)	36.8 (36.5,37.7)
Heart rate, bpm^b^	88 (78,100.8)	87 (78,98)	88 (75,100)	88 (77,102)	89 (80,102)
Systolic pressure, mmHg^c^	126 (114,138)	126 (112,139)	125 (111,136)	128(115,137)	127 (118,140)
Diastolic pressure, mmHg^c^	75 (68,84)	75 (68,84)	756 (68,86)	76 (70,86)	76 (69,82)
**Initial symptoms**					
Fever	172 (81.5%)	44 (83%)	42 (79.2%)	45 (84.9%)	41 (78.8%)
Symptoms of respiratory system	168 (79.6%)	41 (77.4%)	42 (79.2%)	43 (81.1%)	42 (80.8%)
Neuromuscular symptoms	79 (37.4%)	20 (37.7%)	22 (41.5%)	17 (32.1%)	20 (38.5%)
Digestive symptoms	55 (26.1%)	13 (24.5%)	13 (24.5%)	16 (30.2%)	13 (25%)
Onset of symptom to admission, d^d^	10 (8,14)	13 (10,16)	10 (8,14)	10 (7,14)	10 (7,12)^*^
**Comorbidity**					
Diabetes	33 (15.6%)	7 (13.2%)	10 (18.9%)	6 (11.3%)	10 (19.2%)
Hypertension	71 (33.6%)	20 (37.7%)	17 (32.1%)	16 (30.2%)	18 (34.6%)
Coronary heart disease	15 (7.1%)	3 (5.7%)	2 (3.8%)	2 (3.8%)	8 (15.4%)
COPD	6 (2.8%)	0 (0%)	3 (5.7%)	3 (5.7%)	0 (0%)
Cerebrovascular disease	7 (3.3%)	2 (3.8%)	1 (1.9%)	2 (3.8%)	2 (3.8%)
Chronic renal disease	7 (3.3%)	1 (1.9%)	1 (1.9%)	3 (5.7%)	2 (3.8%)
Chronic liver disease	9 (4.3%)	1 (1.9%)	3 (5.7%)	2 (3.8%)	3 (5.8%)
Only one comorbidity	56 (26.5%)	12 (22.6%)	17 (32.1%)	12 (22.6%)	15 (28.8%)
≥2 comorbidities	40 (19%)	10 (18.9%)	8 (15.1%)	10 (18.9%)	12 (23.1%)

*COPD, chronic obstructive pulmonary diseases; Data are Median (IQR) or n (%). The total number of patients with available data: a: n(A) = 53, n(B) = 52, n(C) = 53, n(D) = 51; b: n(A) = 53, n(B) = 53, n(C) = 53, n(D) = 51; c: n(A) = 42, n(B) = 44, n(C) = 46, n(D) = 41; d: n(A) = 53, n(B) = 52, n(C) = 52, n(D) = 52;*

* *P < 0.05 vs. group A. Different laboratory indicators have different data volumes, different alphabets are used to show the number of patients in each group of this indicator*.

We compared the characteristics of patients in the four LDL-C groups on admission. Body temperature, heart rate and blood pressure did not differ among the four groups. The most common symptoms before admission overall were fever (81.5%) and respiratory symptoms (79.6%). There were no significant differences among the groups in terms of initial symptoms. Overall, the most common comorbidities were hypertension (33.6%), followed by diabetes (15.6%) and coronary heart disease (7.1%). Although the proportions of all comorbidities in Group D were higher than those in Group A, there were no significant differences between the two groups (Table 1).

### Comparison of Laboratory Test Results Among the Four Groups

**Figure 2:**
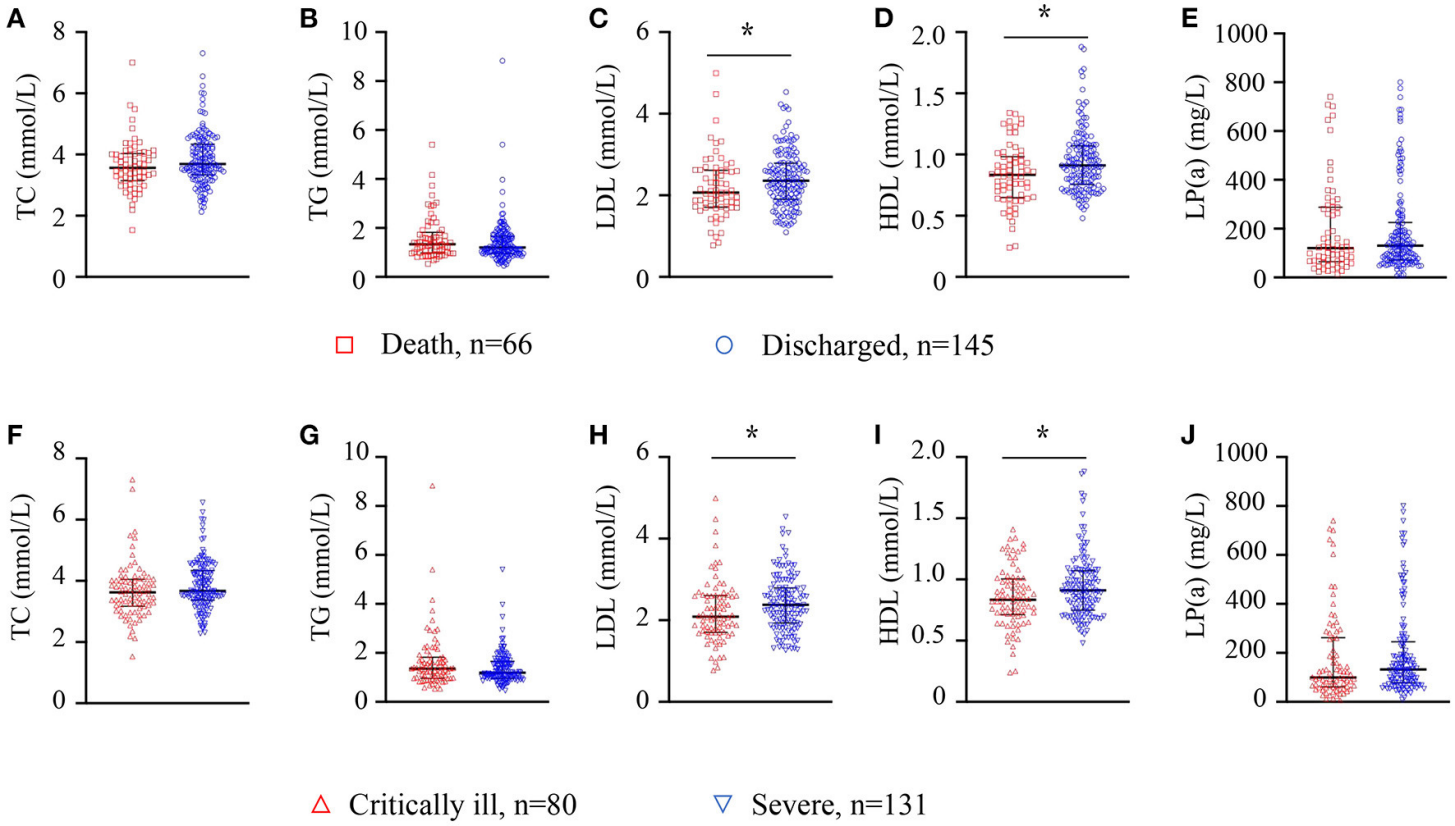
Dyslipidemia was observed in severe COVID-19 patients. Serum TC, TG, LDL, HDL and LP (a) levels are presented from COVID-19 patients in the death (*n* = 66) and discharged groups (*n* = 145) **(A–E)**, or in the critically ill (*n* = 80) and severe groups (*n* = 131) **(F–J)**. Whiskers represent the median (IQR) in the plots. The Mann–Whitney *U*-test was used to compare differences between the groups. TC, total cholesterol; TG, triglyceride; LDL, low-density lipoprotein; HDL, high-density lipoprotein, LP (a), lipoprotein (a). *Means *P*-value < 0.05.

The laboratory test results within 3 days after admission were collected and analyzed. We first analyzed the results of serum lipids and found that levels of TC, triglyceride (TG) and lipoprotein (a) [LP(a)] decreased with LDL-C reduction. However, levels of HDL-C showed no differences among the four groups (Table 2). We further compared the levels of serum lipids between the death and discharged groups and found that the discharged patients had higher levels of LDL-C and HDL-C than did the patients who died. No differences were found in terms of levels of TC, TG or LP(a) between the death and discharged groups (Figures 2A–E). We also compared the levels of serum lipid between the severe and critically ill groups. We found that critically ill patients had lower levels of LDL-C and HDL-C than did severe patients. No differences were found between these two groups in terms of levels of TC, TG or LP(a) (Figures 2F–J). These results suggest the existence of dyslipidemia in severe and critically ill COVID-19 patients.

**Table 2:** Comparison of laboratory test results among four groups.

**Parameters**	**All (*n* = 211)**	**A (*n* = 53)**	**B (*n* = 53)**	**C (*n* = 53)**	**D (*n* = 52)**
**Serum Lipids**					
TC, mmol/L	3.7 (3.3,4.2)	4.6 (4.2,5)	3.9 (3.7,4.1)^*^	3.5 (3.3,3.6)^*^^#^	2.9 (2.6,3.2)^*^^#^^&^
TG, mmol/L	1.2 (1,1.7)	1.5 (1.1,2)	1.4 (1.1,1.7)	1.1 (0.9,1.5)^*^	1.1 (0.9,1.4)^*^^#^
HDL, mmol/L	0.9 (0.7,1)	0.9 (0.8,1.1)	0.9 (0.8,1)	0.9 (0.7,1.1)	0.9 (0.7,1)
Lipoprotein a, mg/L	124.0 (69,253.7)	159 (99,296)	127 (69,254)	124 (68,191)^*^	90.5 (63,145.5)^*^^#^
**Arterial blood gas analysis**^**a**^					
Arterial oxygen saturation, %	94 (88.3,98)	93 (79.5,96)	95 (89.8,98.3)	95.5 (89.8,98)	93.5 (88.8,97)
Arterial partial pressure of oxygen, mmHg	73 (53,94)	71 (44,82)	74.5 (61,110)	84 (59,101)	71 (54,92)
Lactic acid, mmol/L	2.1 (1.6,2.9)	2.2 (1.7,2.8)	2.2 (1.6,3.6)	2.2 (1.8,3.1)	2.1 (1.4,2.4)
**Blood routine**^**b**^					
White blood cell count, × 10^9^/L	5.7 (4.4,8.3)	5.8 (4.7,8.7)	7 (5.2,8.4)	5.5 (4.3,7)	5.3 (4.1,7.9)
Neutrophil count, × 10^9^/L	4.2 (2.9,6.7)	3.9 (3.1,6.9)	5.5 (3,6.9)	3.7 (2.6,5.5)	4.1 (2.6,6.8)
Neutrophil %	75 (62.9,85.8)	72.2 (65.2,83.5)	80.5 (66.1,89.3)	74.4 (61.1,83)	77.1 (64.7,89.6)
Lymphocyte count, × 10^9^/L	0.9 (0.6,1.3)	1 (0.8,1.5)	0.8 (0.5,1.3)^*^	0.9 (0.8,1.4)	0.7 (0.5,1.1)^*^^&^
Lymphocyte %	16.7 (8.5,25.9)	19.6 (9.6,25.5)	12.9 (5.5,23.1)	16.9 (11.1,28.9)	16.2 (6.4,26.7)
Platelet count, × 10^9^/L	200 (154,263)	240 (195,325)	222 (176,285)	185 (148,222)^*^^#^	156.5 (120.3,204.8)^*^^#^
<125	27 (12.9%)	1 (1.9%)	5 (9.4%)	7 (13.2%)^*^	14 (28%)^*^^#^
>350	18 (8.6%)	10 (18.9%)	7 (13.2%)	0 (0%)^*^^#^	1 (2%)^*^
Red blood cell count, × 10^10^/L	4.1 (3.8,4.5)	4.1 (3.7,4.5)	4.1 (4,4.4)	4.2 (3.9,4.6)	4 (3.7,4.3)
**Liver function**					
ALT,U/L	25 (18,40)	25 (18,59)	25 (19,40)	21 (16,37)	29 (20.5,36)
AST,U/L	30 (22,46.5)	29 (22,45)	29 (21,41)	28 (20,45)	39 (27.5,54.3)^*^^#^^&^
Total bilirubin, mol/L	10.9 (8.4,15.5)	12.6 (8.8,16.6)	11.6 (9.7,18.4)	9.7 (7.3,13.5)^*^^#^	10.6 (8.4,15)
Total protein, g/L	60.2 (56.8,64.5)	62.2 (58.6,65.2)	60.8 (57.2,64.4)	59.4 (56.5,65.2)	58.8 (56.3,62.6)
**Kidney function**					
Creatinine, μmol/L	61 (50.5,76)	56 (49,70)	60 (50,73)	62 (52,78)	70.5 (51.8,84.3)
Blood urea nitrogen, nmol/L	4.8 (3.7,6.9)	4.1 (3.7,5.8)	5.2 (3.6,7.2)	5 (3.4,7)	4.9 (3.9,7.6)
Uric acid, μmol/L	240 (200.5,320)	231 (199,293)	243 (214,299)	261 (219,338)	236.5 (188.8,335.5)
EGFR, mL/min	96.6 (89.1,106.8)	96.9 (91.6,106.4)	95.5 (88.9,106.9)	99.1 (88.4,113.2)	94.2 (80.1,101.9)
≤ 90	56 (26.5%)	9 (17%)	15 (28.3%)	14 (26.4%)	18 (34.6%)^*^
**Cardiac injury**					
Creatine kinase, U/L	67 (41.5,117)	65 (44,94)	56 (35,105)	79 (48,119)^#^	80 (52.5,243.8)^#^
>310	21 (10%)	2 (3.8%)	3 (5.7%)	5 (9.4%)	11 (21.2%)^*^^#^
Lactate dehydrogenase, U/L^c^	310 (230,426.3)	305.5 (245.5,375.3)	311 (227,451)	292 (212,427)	355.5 (247,453)
Myoglobin, μg/L^d^	46.9 (31.6,86.1)	51.1 (33,74.3)	47.2 (28.8,87)	40.3 (33.3,69)	57.8 (33.2,86.6)
Creatine kinase-myocardial band isoenzyme, ng/ml^**e**^	1 (0.7,2.1)	1 (0.6,2.1)	1 (0.7,2.3)	1.1 (0.7,1.6)	1.2 (0.6,2.5)
Hypersensitive troponin I, ng/mL^f^	0.006 (0.006,0.023)	0.006 (0.006,0.013)	0.006 (0.006,0.012)	0.006 (0.006,0.029)	0.009 (0.006,0.049)
**Non-specific inflammation index**					
C-reactive protein, mg/L^g^	47.5 (10.9,92.3)	27.7 (6.6,62.9)	65.5 (28.3,96.9)^*^	51 (5.7,89.5)	58.1 (23.5,104.1)^*^
>10,	139 (75.5%)	30 (63.8%)	42 (85.7%)^*^	32 (72.7%)	35 (79.5%)
High-sensitivity C-reactive protein, mg/L^g^	5 (5,5)	5 (5,5)	5 (5,5)	5 (5,5)	5 (5,5)
Procalcitonin, ng/mL^h^	0.08 (0,0.17)	0.056 (0.035,0.102)	0.081 (0.047,0.17)^*^	0.039 (0.05,0.17)	0.049 (0.11,0.29)^*^
**Coagulation function**^**i**^					
D-dimer, μmol/L	0.8 (0.5,2.2)	0.7 (0.5,1.8)	1 (0.5,2.6)	0.6 (0.3,1.8)	0.8 (0.4,2.2)
Prothrombin time activity, %	81.2 (74.4,88)	83.2 (74.9,92.5)	80 (74.3,88.8)	81.4 (74.6,84.9)	78.8 (71.5,87.6)
Activated partial thromboplastin time, s	28.5 (26.1,31)	27.3 (24.9,28.7)	27.7 (26.3,30.7)	29.3 (27.3,32.7)^*^	29.8 (28.1,33.6)^*^^#^
**Cellular immunity**^**j**^					
CD16+56, %	8 (8,21.8)	14.2 (8.8,21.6)	12.5 (6.5,22.1)	14 (9.7,18.5)	14.6 (8.7,22.5)
CD16+56 counts, No./μl	69 (69,179.8)	148 (78,190)	88 (59.5,124.5)	100 (74,223)	103 (64,153.5)
CD19, %	12.3 (12.3,21.8)	17.3 (13.5,21.8)	16.9 (12.2,21.4)	16.4 (12.8,20)	14.9 (10.7,23.5)
CD19 counts, No./μl	84.5 (84.5,202.5)	148 (109.5,245)	127 (68.5,200.5)	141 (103,190)	103 (74.5,144)^*^^&^
CD3, %	55 (55,73.3)	62.9 (55.3,69.9)	66 (51.6,74.6)	68.1 (58.9,75.2)	62.3 (53.8,73.1)
CD3 counts No./μl	311 (311,822.8)	551 (411.5,835)	466 (270,961.5)	582 (436,972)	467 (258.5,694.5)
CD4, %	32.3 (32.3,46.1)	40.1 (33.9,46.2)	40.6 (31.5,48.2)	41 (32.4,46)	38 (31,44.3)
CD4 counts, No./μl	193 (193,510.8)	380 (304,520.5)	275 (151,581.5)^*^	346 (207,576)	266 (162,399)^*^
CD8, %	15.4 (15.4,29)	18.7 (14.9,26.5)	20.6 (15,27.8)	25.1 (16,30.3)	20 (15.6,31.3)
CD8 counts, No./μL	95.5 (95.5,287.3)	173 (121,263)	176 (75,296.5)	215 (126,316)	123 (87,272.5)
CD4/CD8	1.2 (1.2,2.8)	2.1 (1.4,3)	1.9 (1.2,3.2)	1.6 (1.2,2.5)	1.7 (1.2,2.5)

*TC, total cholesterol; TG, triglyceride; HDL, high-density lipoprotein; ALT, alanine aminotransferase; AST, aspartate aminotransferase; EGFR, estimated glomerular filtration rate; Data are Median (IQR) or n (%). The total number of patients with available data: a: n(A) = 26, n(B) = 24, n(C) = 28, n(D) = 36; b: n(A) = 53, n(B) = 53, n(C) = 53, n(D) = 50; c: n(A) = 52, n(B) = 53, n(C) = 53, n(D) = 52; d: n(A) = 47, n(B) = 49, n(C) = 45, n(D) = 39; e: n(A) = 47, n(B) = 49, n(C) = 45, n(D) = 40; f: n(A) = 48, n(B) = 49, n(C) = 46, n(D) = 40; g: n(A) = 47, n(B) = 49, n(C) = 44, n(D) = 44; h: n(A) = 49, n(B) = 49, n(C) = 49, n(D) = 47; i: n(A) = 51, n(B) = 48, n(C) = 48, n(D) = 46; j: n(A) = 43, n(B) = 43, n(C) = 41, n(D) = 43;*

* *P < 0.05 vs. group A;*

# *P < 0.05 vs. group B;*

& *P < 0.05 vs. group C. Different laboratory indicators have different data volumes, different alphabets are used to show the number of patients in each group of this indicator*.

Complete blood count results showed that lymphocyte counts decreased with the reduction in LDL-C levels (median, 1 vs. 0.8 vs. 0.9 vs. 0.7 × 10^9^/L). We further analyzed lymphocyte subsets. CD19 counts in Group D were significantly lower than those in Groups A and C. CD4 counts in Groups D and B were lower than those in Group A. There were no differences in other lymphocyte subsets among the four groups. These results suggest that severe and critically ill COVID-19 patients had immune dysfunction and low LDL-C levels may be associated with the severity of immune dysfunction (Table 2).

The platelet counts across the four groups decreased with the reduction in LDL-C levels (median 240 vs. 222 vs. 185 vs. 165.5 × 10^9^/L). The proportion of patients with thrombocytopenia increased with LDL-C reduction. The proportion of patients with thrombocytosis decreased with LDL-C reduction. As platelets are associated with coagulation function, we analyzed the relevant parameters of coagulation function. There were no differences in D-dimer levels or prothrombin time among the four groups. However, activated partial thromboplastin time increased with the reduction in LDL-C levels. Patients with low LDL-C levels were more prone to have coagulation dysfunction (Table 2).

We analyzed parameters revealing liver function, including alanine aminotransferase (ALT), aspartate aminotransferase (AST), total bilirubin and total protein. Patients in Group D had higher levels of AST than did patients in the other three groups. Total bilirubin levels decreased with the reduction in LDL-C. Regarding renal function tests, there were no differences among the groups in terms of levels of creatinine, urea, uric acid or estimated glomerular filtration rate (eGFR). However, the proportion of patients with reduced eGFR in four groups increased with LDL-C reduction (17 vs. 28.3 vs. 26.4 vs. 34.6%). In terms of cardiac injury biomarkers, levels of creatine kinase (CK) and the proportion of patients with increased CK increased with the reduction in LDL-C. No differences were found for other biomarkers among the four groups, including creatinine kinases MB isoenzyme (CKMB) and hs-TnI. These results suggest that severe and critically ill COVID-19 patients with lower LDL-C levels experienced more severe liver dysfunction, renal dysfunction and cardiac dysfunction on admission (Table 2).

The results of arterial blood gas analysis showed no significant differences in lactic acid levels among the four groups. We then analyzed levels of inflammation biomarkers. Levels of C-reactive protein (CRP) in Groups D and B were significantly higher than those in Group A. And Group B had higher procalcitonin levels. But interestingly, procalcitonin levels of Group D reduced, which may reveal less co-infection of COVID-19 patients. However, white blood cell and neutrophil counts showed no differences among four groups. These results suggest that patients with lower LDL-C levels had more severe inflammation (Table 2).

### Comparison of Treatments and Outcomes Among the Four Groups

Respiratory support was the main treatment for COVID-19 patients. A total of 190 (90%) patients received nasal catheter oxygen inhalation and 79 (37.4%) patients received mask oxygen inhalation. The proportion of patients receiving mask oxygen inhalation increased with LDL-C reduction. The proportion of patients with mechanical ventilation, both invasive and non-invasive, increased with LDL-C reduction. The most common drug therapies were antibiotic treatment (62.2%) and immunoglobulin therapy (58.8%). There were no differences in drug treatments among the four groups, including lipid-lowering therapy. Special treatments such as extracorporeal membrane oxygenation, continuous renal replacement therapy and artificial liver support system were also given during hospitalization. There were no differences among the groups in terms of use of these treatments (Table 3).

**Table 3:** Comparison of treatments and outcomes among four groups.

	**All (*n* = 211)**	**A (*n* = 53)**	**B (*n* = 53)**	**C (*n* = 53)**	**D (*n* = 52)**
**Oxygen therapy**					
Nasal catheter oxygen inhalation	190 (90%)	49 (92.5%)	49 (92.5%)	45 (84.9%)	47 (88.7%)
Mask oxygen inhalation	79 (37.4%)	15 (28.3%)	17 (32.1%)	20 (37.7%)	27 (50.9%)^*^
HFBHTI	21 (10%)	3 (5.7%)	6 (11.3%)	6 (11.3%)	6 (11.3%)
Non-invasive mechanical ventilation	54 (25.6%)	10 (18.9%)	11 (20.8%)	12 (22.6%)	21 (39.6%)^*^^#^
Invasive mechanical ventilation	21 (10%)	3 (5.7%)	2 (3.8%)	5 (9.4%)	11 (20.8%)^*^^#^
**CRRT**	15 (7.1%)	2 (3.8%)	1 (1.9%)	6 (11.3%)	6 (11.3%)
**ECMO**	1 (0.5%)	0 (0%)	0 (0%)	1 (1.9%)	0 (0%)
**Medical treatment**	204 (96.7%)	52 (98.1%)	52 (98.1%)	50 (94.3%)	50 (94.3%)
Antiviral treatment	101 (47.9%)	20 (37.7%)	32 (60.4%)	26 (49.1%)	23 (44.2%)
Antibiotic treatment	146 (69.2%)	33(62.3%)	42 (79.2%)	35 (66.0%)	36 (69.2%)
Antifungal treatment	9 (4.3%)	3 (5.7%)	1 (1.9%)	1 (1.9%)	4 (7.7%)
Glucocorticoids	113 (53.6%)	29 (54.7%)	28 (52.8%)	24 (45.3%)	32 (61.5%)
Immunoglobulin therapy	124 (58.8%)	30 (56.6%)	35 (66%)	25 (47.2%)	34 (64.2%)
Statins	17 (8.1%)	7 (13.2%)	3 (5.7%)	1 (1.9%)	6 (11.3%)
**Complications**					
Shock	36 (17.1%)	4 (7.5%)	10 (18.9%)	10 (18.9%)	12 (22.6%)^*^
Acute cardiac injury	43 (20.4%)	5 (9.4%)	8 (15.1%)	14 (26.4%)^*^	16 (30.2%)^*^
Acute renal injury	20 (9.5%)	3 (5.7%)	4 (7.5%)	6 (11.3%)	7 (13.2%)
Acute liver injury	28 (13.3%)	6 (11.3%)	5 (9.4%)	8 (15.1%)	9 (17%)
Only one complication	45 (21.3%)	11 (20.8%)	13 (24.5%)	7 (13.2%)	14 (26.9%)
≥2 complications	31 (14.7%)	3 (5.7%)	5 (9.4%)	12 (22.6%)^*^	12 (23.1%)^*^
**Admission to ICU**	35 (16.6%)	5 (9.4%)	8 (15.1%)	7 (13.2%)	15 (28.8%)^*^
ICU treatment duration, day^a^	11 (6, 16)	9 (5, 11)	14 (12, 18)	7 (2.5, 12)	9 (6, 17)
**Clinical classification**					
Severe	131 (62.1%)	39 (73.6%)	33 (62.3%)	32 (60.4%)	27 (51.9%)^*^
Critical	80 (37.9%)	14 (26.4%)	20 (37.7%)	21 (39.6%)	25 (48.1%)^*^
**Prognosis**					
Death	66 (31.3%)	12 (22.6%)	15 (28.3%)	18 (34%)	21 (40.4%)
Discharged	145 (68.7%)	41 (77.4%)	38 (71.7%)	35 (66%)	31 (59.6%)
From admission to death, d	4 (4, 10)	7 (5, 8)	5 (3, 10)	8 (5, 13)	8 (6, 9)
From admission to discharge, d	18 (18, 27)	23 (14, 27)	24 (19, 27)	23 (20, 26)	23 (17, 26)

*HFBHTI, high flow breath humidification treatment; CRRT, continuous renal replacement therapy; ECMO, extracorporeal membrane oxygenation; ICU, intensive care unit; IQR, interquartile range. Data are Median (IQR) or n (%). The total number of patients with available data: a: *n*(A) = 5, *n*(B) = 8, *n*(C) = 7, *n*(D) = 15;*

* **P* < 0.05 vs. group A;*

# *P < 0.05 vs. group B. Different laboratory indicators have different data volumes, different alphabets are used to show the number of patients in each group of this indicator*.

Acute cardiac injury (20.4%) and shock (17.1%) were the most common complications of severe COVID-19. The percentage of patients with shock (7.5 vs. 18.9 vs. 18.95 vs. 22.6%) and acute cardiac injury (9.4 vs. 15.1 vs. 26.4 vs. 30.2%) increased with LDL-C reduction. The proportion of patients with acute liver injury or renal injury also increased with LDL-C reduction, although there were no differences among the four groups. We also found that the number of patients with more than one complication increased with LDL-C reduction, which might suggest worse prognosis (Table 3).

There were 35 (16.6%) patients admitted to the ICU within 30 days of admission. Overall ICU treatment duration was 11 (6, 16) [median, (IQR)] days. With the reduction in LDL-C levels, ICU care increased (9.4 vs. 15.1 vs. 13.2 vs. 28.8%). However, there were no differences in ICU treatment duration among the four groups.

The proportion of critically ill patients increased with LDL-C reduction (26.4 vs. 37.7 vs. 39.6 vs. 48.1%). The death cases accounted for 31.3%, and the discharged patients accounted for 68.7%. Mortality across the four groups increased with LDL-C level reduction (22.6 vs. 28.3 vs. 34 vs. 40.4%), although there were no significant differences. The median (IQR) days of hospitalization of death cases were 4 (4,10) days. The median (IQR) days of hospitalization for discharged patients were 18 (18,27) days. There were no differences among the four groups in terms of days of hospitalization for death and discharged cases (Table 3).

### Relationships of CRP, Lymphocyte With LDL-C Levels

**Figure 3:**
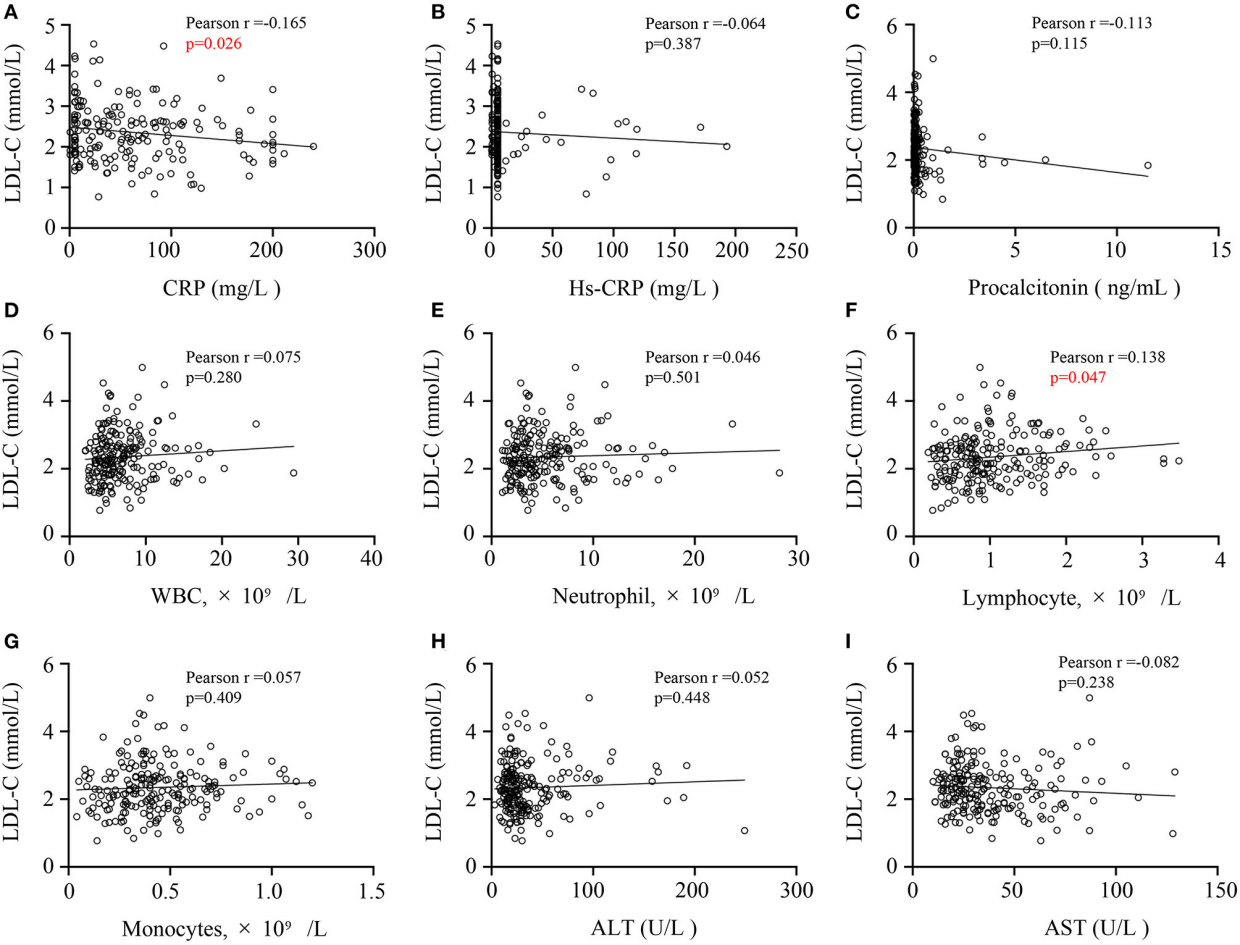
Correlations of CRP levels, CRP levels **(A)**, hs-CRP levels **(B)**, procalcitonin levels **(C)**, WBC counts **(D)**, neutrophil counts **(E)**, lymphocyte counts **(F)**, monocytes count **(G)**, ALT **(H)** or AST levels **(I)** and LDL-C levels in COVID-19 patients. A Pearson correlation analysis was used. CRP, C-reactive protein; hs-CRP, high-sensitivity C-reactive protein; WBC, white blood cell; ALT, alanine aminotransferase; AST, aspartate aminotransferase; LDL-C, low-density lipoprotein cholesterol.

We conducted Pearson correlation analysis to figure out the relationships between LDL-C levels and other laboratory indicators. Serum LDL-C levels of COVID-19 patients was negatively correlated with CRP level (*r* = −0.165, *P* = 0.026, Figure 3A), but positively correlated with lymphocyte count (*r* = 0.138, *P* = 0.047, Figure 3F). Besides, the level of LDL-C had no correlation with other laboratory indicators such as hs-CRP (Figure 3B), procalcitonin (Figure 3C), white blood cell counts (Figure 3D), neutrophil counts (Figure 3E) and monocytes counts (Figure 3G). And We also tested the relationship between LDL-C and AST or ALT (Figures 3H,I), which revealed the liver function. But we found no correlation between them, suggesting that liver function damage may not be the main factor in the reduction of LDL-C levels. These results may indicate that the decrease in LDL-C levels may be related to inflammation and immune dysfunction in COVID-19 patients.

### Hazard Ratio for Hospitalization Death, Cardiac Injury and ICU Treatment of COVID-19

To determine the relationship between LDL-C level and prognosis of severe and critically ill COVID-19 patients, we made proportional hazard models of LDL-C as a continuous variable or a categorical variable. We defined hospitalization death as the primary end point and admission to ICU and cardiac injury as the secondary end point. These models were adjusted for age, sex and comorbidities, including hypertension, diabetes, coronary heart disease, COPD, cerebrovascular disease, chronic renal disease and chronic liver disease.

**Figure 4:**
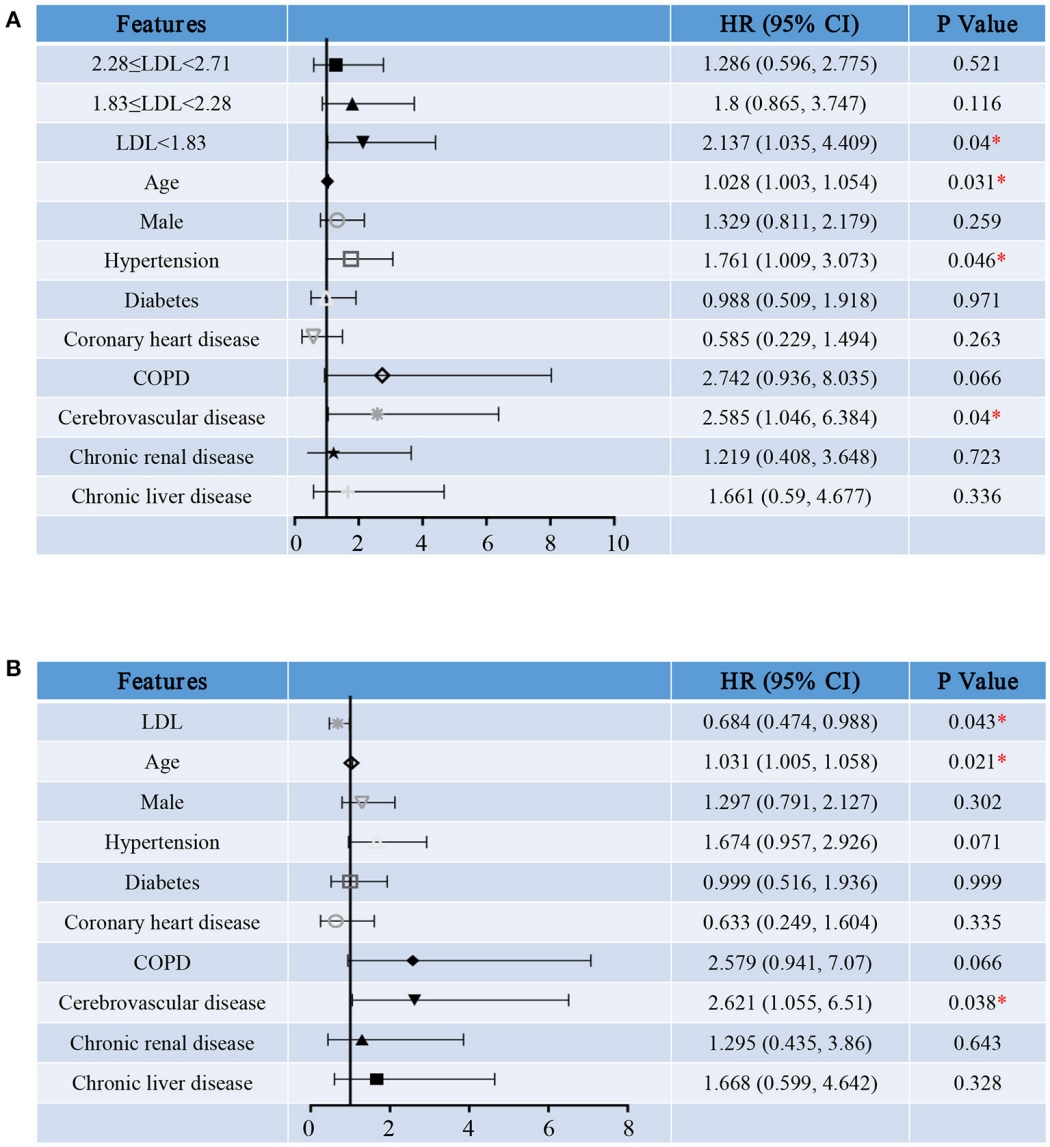
Hazard ratio for hospitalization death of severe COVID-19 patients. Shown in the figure are the hazards ratio (HR) and the 95% confidence interval (95%CI) for the risk factors of hospitalization death after disease onset with treating LDL-C levels as a categorical variable **(A)** or a continuous variable **(B)**. The model has been adjusted with age, sex and comorbidities. *Means the *P*-value <0.05. The scale bar indicates the HR and 95%CI. LDL, low-density lipoprotein; COPD, chronic obstructive pulmonary diseases.

**Figure 5:**
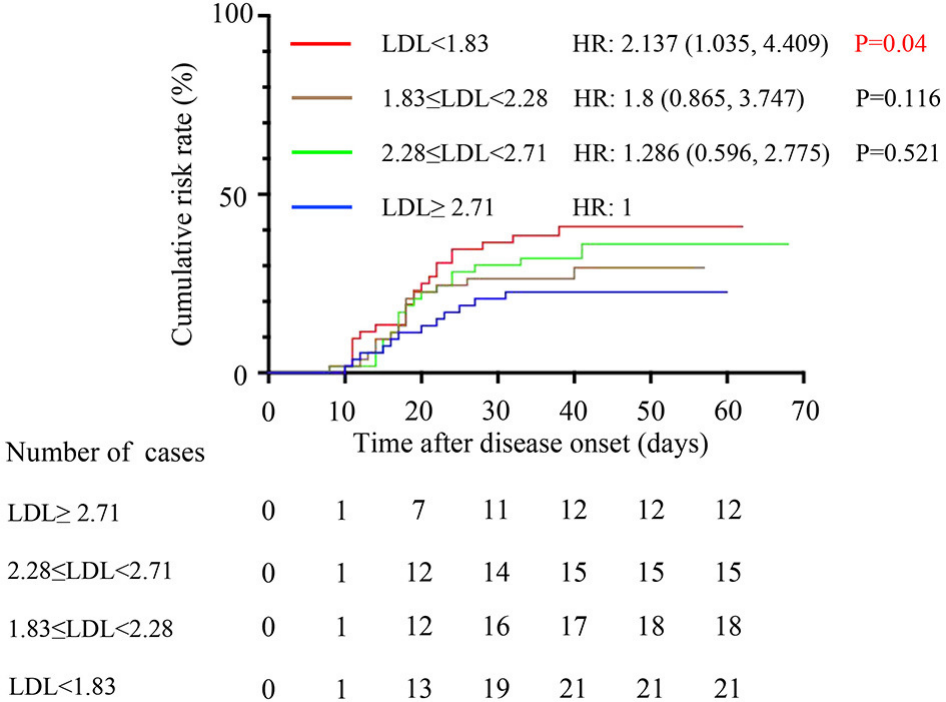
Comparison of the time-dependent risk of hospitalization death. The cumulative hospitalization death risk after disease onset in group A (blue curve), group B (green curve), group C (brown curve) and group D (red curve). The model was adjusted for age, sex and comorbidities. LDL, low-density lipoprotein; HR, hazards ratio; CI, confidence interval.

**Figure 6:**
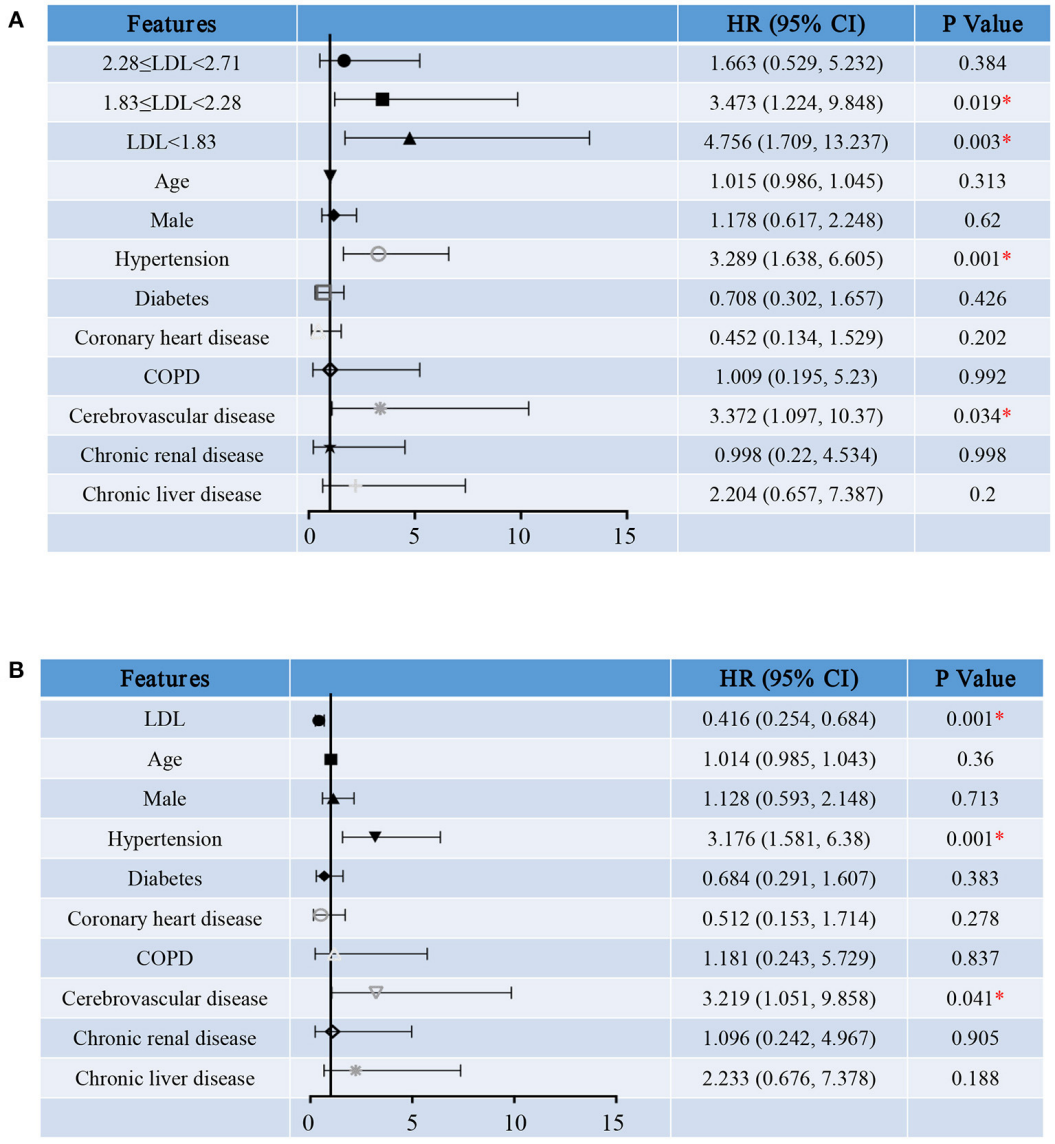
Hazard Ratio for cardiac injury of severe COVID-19 patients. Shown in the figure are the hazards ratio (HR) and the 95% confidence interval (95%CI) for the risk factors of cardiac injury after disease onset with treating LDL-C levels as a categorical variable **(A)** or a continuous variable **(B)**. The model has been adjusted with age, sex and comorbidities. *Means the *P*-value <0.05. The scale bar indicates the HR and 95%CI. LDL, low-density lipoprotein; COPD, chronic obstructive pulmonary diseases.

**Figure 7:**
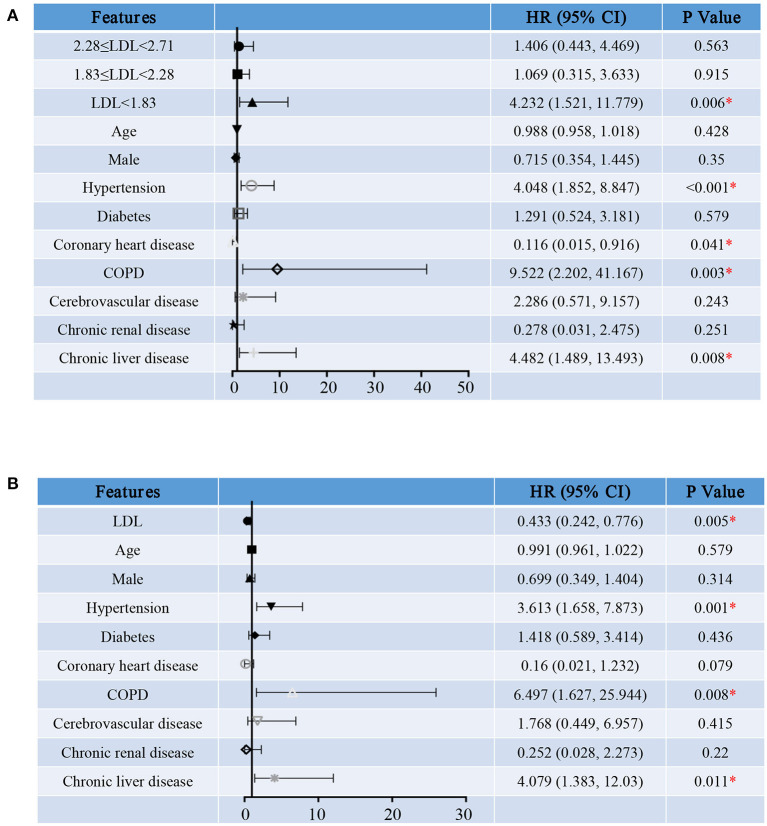
Hazard Ratio for ICU admission of severe COVID-19 patients. Shown in the figure are the hazards ratio (HR) and the 95% confidence interval (95%CI) for the risk factors of ICU admission after disease onset with treating LDL-C levels as a categorical variable **(A)** or a continuous variable **(B)**. The model has been adjusted with age, sex and comorbidities. *Means the *P*-value <0.05. The scale bar indicates the HR and 95%CI. LDL, low-density lipoprotein; COPD, chronic obstructive pulmonary diseases.

We first treated LDL-C as a categorical variable in proportional hazards models. Compared to group A, the risk of hospitalization death in group D [HR (95% CI), 2.137 (1.035, 4.409), *P* = 0.04] was significantly increased (Figure 4A). In addition, survival curve with hospitalization death as the end events was made to observe the relationship between LDL-C level and the risk of hospitalization death more intuitively and clearly (Figure 5). Besides, the risk for cardiac injury in group C [HR (95% CI), 3.473 (1.224, 9.848), *P* = 0.019] and D [HR (95% CI), 4.756 (1.709, 13.237), *P* = 0.003] patients was significantly increased (Figure 6A). Although the risk of admission to ICU for patients in group B and C was not statistically significant compared with group A, the risk of patients in group D [HR (95% CI), 4.232 (1.521, 11.779), *P* = 0.006] significantly increased (Figure 7A). Consistent with previous studies, the regression model in our study also suggests that age, hypertension and cerebrovascular disease are independent risk factors for hospitalization death in patients with severe COVID-19 (Figure 4A) (14). In addition, patients with previous hypertension and cerebrovascular disease are more likely to have cardiac injury during hospitalization (Figure 6A). Moreover, patients with previous hypertension, coronary heart disease, COPD and chronic liver disease are at higher risk of ICU admission during hospitalization (Figure 7A).

Then, we reconstructed the regression model treating serum LDL-C level as a continuous variable. As the level of LDL-C increases, the risk for hospitalization death [HR (95% CI), 0.684 (0.474, 0.988), *P* = 0.043, Figure 4B], cardiac injury [HR (95% CI), 0.416 (0.254, 0.684), *P* = 0.001, Figure 6B] and ICU admission [HR (95% CI), 0.433 (0.242, 0.776), *P* = 0.005, Figure 7B] of COVID-19 patients are reduced. These results further indicate that as the serum LDL-C level decreases, the risks of hospitalization death, cardiac injury and ICU admission to COVID-19 patients are gradually increasing. Decreased LDL-C level indicates poor prognosis of severe and critical COVID-19 patients.

## Discussion

Dyslipidemia was found in severe and critical COVID-19 patients. However, the major symptoms before admission, characteristics on admission and previous comorbidities of severe and critically ill COVID-19 patients showed no differences among the four groups. Patients with lower LDL-C levels were more likely to have immune and inflammation dysfunction, renal dysfunction, liver dysfunction and cardiac dysfunction than patients with higher LDL-C levels on admission. With the reduction in LDL-C levels, the proportion of patients with complications including shock and acute cardiac injury, admission to ICU and use of mechanical ventilation increased. In addition, mortality increased as the LDL-C levels decreased. Serum LDL-C level is related to CRP level and lymphocyte count. Proportional hazards models showed that low LDL-C levels may be associated with increased risk of hospitalization death, cardiac injury and admission to ICU, indicating the prognostic role of LDL-C levels.

As was previously reported, dyslipidemia in COVID-19 was also observed in our study. LDL-C levels in the death and critically ill groups were significantly lower than those in the discharged and severe groups. These were not significantly related to treatments because there were no differences among the four groups in terms of medical treatments and serum lipid levels were collected within 3 days after admission. There is no difference in the comorbidities of the four groups of patients, such as coronary heart disease. The proportion of patients with statins treatment has no significant difference among four groups. We have reasons to speculate that the lipid-lowering therapy is not the cause of the dyslipidemia in patients with COVID-19. Besides, a report from China showed that in-hospital use of statins is associated with a reduced risk of mortality among patients with COVID-19 (16). Consistent with previous studies, we found that mortality across the four groups increased with the reduction in LDL-C levels, although without significant differences. Moreover, low LDL-C levels increased the risk for hospitalization death, cardiac injury and admission to ICU.

Low LDL-C level was found in several diseases, including malignancy, malabsorption, anemia, chronic infections and infestations, and severe illness in hospitalized patients (17). The reasons for dyslipidemia remain unclear. There are several speculations about the causes of dyslipidemia. First, there was a negative correlation between TC levels and the incidence of hospital-acquired infections (18). In general, infection will reduce the levels of TC, LDL-C and HDL-C, while the content of TG will increase. However, the level of TG will also decrease as the nutritional status decrease due to infection. Levels of LDL-C, TC and HDL inversely correlated with CRP levels (11). These studies suggest that dyslipidemia may be associated with infection. In our study, levels of CRP and procalcitonin in Group D were significantly higher than those in Group A. However, there were no differences among the other groups in terms of infection biomarkers. Besides, the results of Pearson correlation analysis showed that serum LDL-C levels of COVID-19 patients was negatively correlated with CRP level, but has nothing to do with hs-CRP, procalcitonin. These findings suggest that dyslipidemia in COVID-19 patients cannot be explained solely by co-infection. Second, lipids were reported to be indicators of liver function changes in patients with hepatitis B (19). The dysfunction of liver is related to the decrease of LDL-C. In addition, some antiviral treatment may have adverse effects like liver dysfunction (20). In our study, patients with low LDL-C levels were more likely to have liver dysfunction on admission. But our results showed that serum LDL-C levels of COVID-19 patients had no significant correlation with ALT and AST levels, revealing that liver dysfunction may be not the main cause of decreased LDL-C levels. Third, cholesterol is essential for viral replication because it constitutes the main component of the viral membrane. Metabolism of lipids was reported to be necessary for the replication of various viruses (21). It has been reported that remodeling of host lipid metabolism is significantly related to the reproduction of human pathogenic coronavirus (22). These findings suggest that dyslipidemia in COVID-19 may corelate with SARS-CoV-2 replication. Fourth, exudates were found in lung pathological examinations of COVID-19 patients (23), and the exudate contained substances such as cholesterol and protein (24). We speculate that the decreased levels of LDL-C might be associated with increased tissue permeability in COVID-19 patients. Fifth, a recent study suggested that changes in metabolites and blood lipids in patients with COVID-19 had a clear correlation with the disease process, suggesting that the development of COVID-19 affects systemic metabolism in COVID-19 patients (25). Lipids are important substances for energy metabolism. The altered energy metabolism may be another reason for dyslipidemia in COVID-19 patients.

In addition to the reduction in LDL-C levels, we also found that HDL-C and LP (a) levels in critically ill or dead COVID-19 patients were reduced. Decreased levels of HDL-C and LP(a) are related to the severity of COVID-19 (26). And HDL-C level was negatively correlated with CRP level in COVID-19 patients (9). Low HDL-C levels may indicate a serious infection. Besides, the decrease in HDL-C levels may be related to the increase in smaller LDL-C particles (27). Several cytokines and inflammatory mediators overexpressed during COVID-19 may directly or indirectly inhibit the activity of lipoprotein lipase (LPL), a key enzyme in lipid metabolism (28). Reduced LPL activity may lead to low HDL-C levels. In addition, increased cholesteryl ester transfer protein (CETP) may lead to a drop in HDL-C levels and an increase of small LDL particles (29, 30). CETP activity has been reported to be associated with poor prognosis of infection (29, 30). These studies may partially explain the reasons for the decreased levels of HDL-C. The mechanism of hypolipidemia caused by COVID-19 needs further researches. HDL-C is an inflammatory mediator that buffers toxic molecules by absorbing them and transporting them to the liver for clearance. Low levels of HDL-C may cause abnormal innate immune function, which is the first-line defense mechanism against COVID-19. Studies have shown that HDL-C can regulate innate and adaptive immunity, thereby increasing resistance to viral infections (31). In addition, a recent study showed that HDL-C has antiviral activity against SARS-CoV-2 (32). Decreased HDL-C levels during hospitalization have been reported as a sign of poor prognosis for COVID-19 patients (27). These studies suggested the negative prognostic role of HDL-C in COVID-19 patients, which needs further researches.

LDL particles are heterogeneous and classified into two patterns, large buoyant LDL (lbLDL) and small dense LDL (sdLDL) based on their plasma phenotypes. Compared to lbLDL, the sdLDL particles are cholesterol-depleted, produced by enzymatic reactions in the liver and blood circulation (33). SdLDL is considered to be related to some harmful effects of LDL. We did not analyze the situation of different LDL particles in this study. However, the decrease in HDL-C levels was thought to be related to the increase in sdLDL particles (27). The role of different LDL particles in COVID-19 needs more research to further explain. In addition, the severe inflammatory reaction process in COVID-19 patients is always accompanied by oxidative stress. The oxidative stress and inflammation of endothelial cells, the targets of SARS-CoV-2 and the pro-inflammatory cytokines released during COVID-19 infection, are activated (34). Oxidative stress is manifested by excessive production ROS and oxidized LDL (oxLDL) particles. The production of oxLDL may cause the activation of endothelial cells and macrophages, and further lead to endothelial damage, and even atherosclerotic plaque instability, which may be one of the causes of cardiac injury (35). The important role of oxLDL in atherosclerosis may also explain the worse prognosis of COVID-19 patients with elder age and coronary artery disease. Therefore, some research suggests to improve COVID-19 by regulating the production of oxLDL (36). In this study, the relationship between oxLDL and poor prognosis has not been discussed too much, which requires further exploration.

We noticed that the mortality of severe and critically ill COVID-19 patients increased with the reduction in LDL-C levels. Nevertheless, there were no significant differences among groups in terms of mortality, which may be due to the number of cases in our study. However, decreased LDL-C levels indicate increased risk for hospitalization death. It has been reported that sex is a risk factor for mortality of COVID-19 and male patients were more likely to have poor prognosis (37). In our study, the proportion of male patients increased with the reduction in LDL-C, which may be associated with the increased mortality of patients with low LDL-C levels. In addition, patients in Group D had higher levels of CRP and procalcitonin, which might suggest secondary coinfection. Coinfection was reported to be a risk factor for poor prognosis of COVID-19 (38). Besides, the results of Pearson correlation analysis showed that serum LDL-C levels of COVID-19 patients was negatively correlated with CRP level. These findings suggest that higher levels of CRP might result in poor prognosis in patients with low LDL-C levels. We also noticed that patients with lower LDL-C levels were more likely to have coagulation dysfunction. Coagulation dysfunction was common in COVID-19 patients and may cause complications such as deep vein thrombosis and pulmonary embolism (39). Vascular endothelial injury and thrombosis are considered to be the pathophysiological basis of COVID-19 (40). Severe patients usually had worse coagulation function (41). The increased mortality of patients with low LDL-C levels might be related to severe coagulation dysfunction.

Lymphocyte counts increased with LDL-C reduction. CD19 and CD4 counts were significantly lower in Group D than in Group A. These results suggest worse immune dysfunction in patients with low LDL-C levels, which has been recognized as an important factor in the occurrence and development of COVID-19 since the beginning of the epidemic (42). In addition, the results of Pearson correlation analysis showed that serum LDL-C levels of COVID-19 patients was positively correlated with lymphocyte count. Previous studies showed that lymphopenia was common in COVID-19 patients and the extent of lymphopenia was associated with the severity of COVID-19 (43). These findings suggest that worse immune dysfunction may be the reason for increased mortality in patients with low LDL-C levels. Complications such as cardiac injury and shock were reported to be risk factors for mortality (14). In the present study, the proportion of patients with cardiac injury, shock or more than one complication increased with LDL-C reduction, which may result in poor prognosis of patients with low LDL-C levels.

Increased LDL-C levels can usually increase the risk of cardiovascular disease (CVD) events, while lower levels can reduce the incidence of CVD events (44). Lowering LDL-C levels has been recommended in the secondary prevention of CVD (45). However, in our study, we found that low levels of LDL-C increased the risk for cardiac injury during hospitalization. But our results can only show that decreased LDL-C levels may indicate an increased risk of cardiac damage, but cannot show that cardiac damage is caused by decreased LDL-C levels. Serum LDL-C levels of COVID-19 patients was negatively correlated with CRP level, but positively correlated with lymphocyte count. And patients in the low LDL-C groups had higher levels of inflammation and immune dysfunction, which were reported to be related to myocardial damage (46). The physiological and pathological mechanisms of the increased cardiac injury risk by low LDL-C may be very complicated and requires further study. In addition, we do not recommend COVID-19 patients to stop using statins and other lipid-lowering drugs. The treatment of statins during hospitalization has been reported to reduce the risk of death in COVID-19 patients (16).

There are several limitations to our study. First, this was a single-center retrospective study. We analyzed only laboratory results within 3 days after admission. The dynamic changes in various biomarkers during hospitalization should be further analyzed. Second, this was only a descriptive study. The mechanisms underlying the relationship between prognosis and dyslipidemia of COVID-19 require further research. Besides, we enrolled only 211 cases in this study which is very small. More data are needed to further explore the relationship between LDL-C levels and prognosis of COVID-19. Third, patients who were still hospitalized or transferred to another hospital 30 days after admission were not included, and this may have affected the results. Fourth, we did not exclude patients receiving statins, which may lead to low LDL-C levels. Fifth, the level of serum LDL-C is usually calculated by Friedewald formula in clinical practice. The role of LP(a) has not been neglected, so our data cannot truly reflect the level of serum LDL-C. But our research still has clinical value. Finally, due to the limited data collected, we ignored the impact of medication during hospitalization on the prognosis of patients, and did not analyze the medications used by patients that may have an impact on the results of the study.

## Conclusion

Reduced LDL-C levels were observed in severe and critically ill COVID-19 patients. LDL-C levels may be negatively correlated with COVID-19 severity. Decreased LDL-C level indicates poor prognosis of severe and critical COVID-19 patients.

## Data Availability Statement

The original contributions presented in the study are included in the article/supplementary material, further inquiries can be directed to the corresponding author/s.

## Ethics Statement

This retrospective study was approved by the Ethics Commission of Renmin Hospital of Wuhan University.

## Author Contributions

MZ, ZL, HH, JZ, and JW designed study, collected and analyzed data, and wrote manuscript. JL and YX collected and reviewed clinical, laboratory, and radiological data. JY and ZW performed statistical analysis. DY reviewed, interpreted, and checked clinical data. JW and MW edited manuscript and supervised the study. All authors contributed to the article and approved the submitted version.

## Conflict of Interest

The authors declare that the research was conducted in the absence of any commercial or financial relationships that could be construed as a potential conflict of interest.

## References

[1] Organization WH. WHO characterizes COVID-19 as a pandemic (2020). Available online at: https://www.who.int/emergencies/diseases/novel-coronavirus-2019/events-as-they-happen (accessed on March 11, 2020).

[2] Organization WH. Summary of probable SARS cases with onset of illness from 1 November 2002 to 31 July 2003 (2003). Available online at: https://www.who.int/csr/sars/country/table2004_04_21/en/ (accessed on December 31, 2003).

[3] Organization WH. MERS Situation Update. (2019). Available online at: https://applications.emro.who.int/docs/EMRPUB-CSR-241-2019-EN.pdf?ua=1andua=1andua=1 (accessed November 30, 2019).

[4] Sanders JM Monogue ML Jodlowski TZ Cutrell JB. Pharmacologic treatments for coronavirus disease 2019 (COVID-19): a review. JAMA. (2020) 323:1824–36. 10.1001/jama.2020.6019 32282022

[5] Guan WJ Liang WH Zhao Y Liang HR Chen ZS Li YM. Comorbidity and its impact on 1590 patients with COVID-19 in China: a nationwide analysis. Eur Respir J. (2020) 55:2000547. 10.1183/13993003.00547-2020 32217650 PMC7098485

[6] Zhu L She ZG Cheng X Qin JJ Zhang XJ Cai J. Association of blood glucose control and outcomes in patients with COVID-19 and pre-existing type 2 diabetes. Cell Metab. (2020) 31:1068–77.e1063. 10.1016/j.cmet.2020.04.021 32369736 PMC7252168

[7] Song SZ Liu HY Shen H Yuan B Dong ZN Jia XW. [Comparison of serum biochemical features between SARS and other viral pneumonias]. Zhongguo Wei Zhong Bing Ji Jiu Yi Xue. (2004) 16:664–6. 15535901

[8] Wu Q Zhou L Sun X Yan Z Hu C Wu J. Altered lipid metabolism in recovered SARS patients twelve years after infection. Sci Rep. (2017) 7:9110. 10.1038/s41598-017-09536-z 28831119 PMC5567209

[9] Fan J Wang H Ye G Cao X Xu X Tan W. Letter to the Editor: Low-density lipoprotein is a potential predictor of poor prognosis in patients with coronavirus disease 2019. Metabolism. (2020) 107:154243. 10.1016/j.metabol.2020.154243 32320740 PMC7166305

[10] Hu X Chen D Wu L He G Ye W. Declined serum high density lipoprotein cholesterol is associated with the severity of COVID-19 infection. Clin Chim Acta. (2020) 510:105–10. 10.1016/j.cca.2020.07.015 32653486 PMC7350883

[11] Wei X Zeng W Su J Wan H Yu X Cao X. Hypolipidemia is associated with the severity of COVID-19. J Clin Lipidol. (2020) 14:297–304. 10.1016/j.jacl.2020.04.008 32430154 PMC7192140

[12] Huang W Li C Wang Z Wang H Zhou N Jiang J. Decreased serum albumin level indicates poor prognosis of COVID-19 patients: hepatic injury analysis from 2,623 hospitalized cases. Sci China Life Sci. (2020) 63:1678–87. 10.1007/s11427-020-1733-4 32567003 PMC7306450

[13] China NHC, o.t.P.s.R.o. The Diagnosis Treatment Guidelines of Pneumonia Caused by Novel Coronavirus (6th trial edition). (2020). Available online at: http://www.nhc.gov.cn/yzygj/s7652m/202002/54e1ad5c2aac45c19eb541799bf637e9.shtml (accessed February 19, 2020).

[14] Zhao M Wang M Zhang J Gu J Zhang P Xu Y. Comparison of clinical characteristics and outcomes of patients with coronavirus disease 2019 at different ages. Aging. (2020) 12:10070–86. 10.18632/aging.103298 32499448 PMC7346026

[15] Shi S Qin M Shen B Cai Y Liu T Yang F. Association of cardiac injury with mortality in hospitalized patients with COVID-19 in Wuhan, China. JAMA Cardiol. (2020) 5:802–10. 10.1001/jamacardio.2020.0950 32211816 PMC7097841

[16] Zhang XJ Qin JJ Cheng X Shen L Zhao YC Yuan Y. In-hospital use of statins is associated with a reduced risk of mortality among individuals with COVID-19. Cell Metab. (2020) 32:176–87.e174. 10.1016/j.cmet.2020.06.015 32592657 PMC7311917

[17] Durrington P. Dyslipidaemia. Lancet. (2003) 362:717–31. 10.1016/s0140-6736(03)14234-1 12957096

[18] Iribarren C Jacobs DR Jr Sidney S Claxton AJ Feingold KR. Cohort study of serum total cholesterol and in-hospital incidence of infectious diseases. Epidemiol Infect. (1998) 121:335–47. 10.1017/s0950268898001435 9825784 PMC2809530

[19] Cao WJ Wang TT Gao YF Wang YQ Bao T Zou GZ. Serum lipid metabolic derangement is associated with disease progression during chronic HBV infection. Clin Lab. (2019) 65. 10.7754/Clin.Lab.2019.190525 31850701

[20] Zhao M Zhang J Li H Luo Z Ye J Xu Y. Recent progress of antiviral therapy for coronavirus disease 2019. Eur J Pharmacol. (2021) 890:173646. 10.1016/j.ejphar.2020.173646 33190802 PMC7584884

[21] Heaton NS Randall G. Multifaceted roles for lipids in viral infection. Trends Microbiol. (2011) 19:368–375. 10.1016/j.tim.2011.03.007 21530270 PMC3130080

[22] Yan B Chu H Yang D Sze KH Lai PM Yuan S. Characterization of the lipidomic profile of human coronavirus-infected cells: implications for lipid metabolism remodeling upon coronavirus replication. Viruses. (2019) 11:73. 10.3390/v11010073 30654597 PMC6357182

[23] Tian S Hu W Niu L Liu H Xu H Xiao SY. Pulmonary pathology of early-phase 2019 novel coronavirus (Covid-19) pneumonia in two patients with lung cancer. J Thorac Oncol. (2020) 15:700–4. 10.1016/j.jtho.2020.02.010 32114094 PMC7128866

[24] Sahn SA. The differential diagnosis of pleural effusions. West J Med. (1982) 137:99–108. 6182697 PMC1274018

[25] Wu D Shu T Yang X Song JX Zhang M Yao C. Plasma metabolomic and lipidomic alterations associated with COVID-19. National Sci Rev. (2020) 7:1157–68. 10.1093/nsr/nwaa086 PMC7197563 34676128

[26] Hilser JR Han Y Biswas S Gukasyan J Cai Z Zhu R. Association of serum HDL cholesterol and apolipoprotein A1 levels with risk of severe SARS-CoV-2 infection. J Lipid Res. (2021) 62:100061. 10.1016/j.jlr.2021.100061 33667465 PMC7923911

[27] Masana L Correig E Ibarretxe D Anoro E Arroyo JA Jeric ó C. Low HDL and high triglycerides predict COVID-19 severity. Sci Rep. (2021) 11:7217. 10.1038/s41598-021-86747-5 33785815 PMC8010012

[28] Ng PC Ang IL Chiu RW Li K Lam HS Wong RP. Host-response biomarkers for diagnosis of late-onset septicemia and necrotizing enterocolitis in preterm infants. J Clin Invest. (2010) 120:2989–3000. 10.1172/jci40196 20592468 PMC2912182

[29] Trinder M Genga KR Kong HJ Blauw LL Lo C Li X. Cholesteryl Ester Transfer Protein Influences High-Density Lipoprotein Levels and Survival in Sepsis. Am J Respir Crit Care Med. (2019) 199:854–62. 10.1164/rccm.201806-1157OC 30321485

[30] Trinder M Wang Y Madsen CM Ponomarev T Bohunek L Daisely BA. Inhibition of cholesteryl ester transfer protein preserves high-density lipoprotein cholesterol and improves survival in sepsis. Circulation. (2021) 143:921–34. 10.1161/circulationaha.120.048568 33228395

[31] Catapano AL Pirillo A Bonacina F Norata GD. HDL in innate and adaptive immunity. Cardiovasc Res. (2014) 103:372–83. 10.1093/cvr/cvu150 24935428

[32] Cho KH Kim JR Lee IC Kwon HJ. Native high-density lipoproteins (HDL) with higher paraoxonase exerts a potent antiviral effect against SARS-CoV-2 (COVID-19), while glycated HDL lost the antiviral activity. Antioxidants. (2021) 10:209. 10.3390/antiox10020209 33535459 PMC7912765

[33] Alizadeh-Fanalou S Nazarizadeh A Alian F Faraji P Sorori B Khosravi M. Small dense low-density lipoprotein-lowering agents. Biol Chem. (2020) 401:1101–21. 10.1515/hsz-2019-0426 32427116

[34] Varga Z Flammer AJ Steiger P Haberecker M Andermatt R Zinkernagel AS. Endothelial cell infection and endotheliitis in COVID-19. Lancet. (2020) 395:1417–8. 10.1016/s0140-6736(20)30937-5 32325026 PMC7172722

[35] Poznyak AV Nikiforov NG Markin AM Kashirskikh DA Myasoedova VA Gerasimova EV. Overview of OxLDL and its impact on cardiovascular health: focus on atherosclerosis. Front Pharmacol. (2020) 11:613780. 10.3389/fphar.2020.613780 33510639 PMC7836017

[36] Erol A. Role of oxidized LDL-induced “trained macrophages” in the pathogenesis of COVID-19 and benefits of pioglitazone: A hypothesis. Diabetes Metab Syndr. (2020) 14:713–714. 10.1016/j.dsx.2020.05.007 32470851 PMC7214326

[37] Qin L Li X Shi J Yu M Wang K Tao Y. Gendered effects on inflammation reaction and outcome of COVID-19 patients in Wuhan. J Med Virol. (2020) 92:2684–92. 10.1002/jmv.26137 32497297 PMC7300463

[38] Zhang G Hu C Luo L Fang F Chen Y Li J. Clinical features and short-term outcomes of 221 patients with COVID-19 in Wuhan, China. J Clin Virol. (2020) 127:104364. 10.1016/j.jcv.2020.104364 32311650 PMC7194884

[39] Poggiali E Bastoni D Ioannilli E Vercelli A Magnacavallo A. Deep vein thrombosis and pulmonary embolism: two complications of COVID-19 pneumonia? Eur J Case Rep Intern Med. (2020) 7:001646. 10.12890/2020_001646 32399449 PMC7213837

[40] Magro C Mulvey JJ Berlin D Nuovo G Salvatore S Harp J. Complement associated microvascular injury and thrombosis in the pathogenesis of severe COVID-19 infection: a report of five cases. Transl Res. (2020) 220:1–13. 10.1016/j.trsl.2020.04.007 32299776 PMC7158248

[41] Zou Y Guo H Zhang Y Zhang Z Liu Y Wang J. Analysis of coagulation parameters in patients with COVID-19 in Shanghai, China. Biosci Trends. (2020) 14:285–9. 10.5582/bst.2020.03086 32350161

[42] Mehta P McAuley DF Brown M Sanchez E Tattersall RS Manson JJ. COVID-19: consider cytokine storm syndromes and immunosuppression. Lancet. (2020) 395:1033–34. 10.1016/s0140-6736(20)30628-0 32192578 PMC7270045

[43] Liu J Li S Liu J Liang B Wang X Wang H. Longitudinal characteristics of lymphocyte responses and cytokine profiles in the peripheral blood of SARS-CoV-2 infected patients. EBioMedicine. (2020) 55:102763. 10.1016/j.ebiom.2020.102763 32361250 PMC7165294

[44] Ference BA Ginsberg HN Graham I Ray KK Packard CJ Bruckert E. Low-density lipoproteins cause atherosclerotic cardiovascular disease. 1. Evidence from genetic, epidemiologic, clinical studies. A consensus statement from the European Atherosclerosis Society Consensus Panel. Eur Heart J. (2017) 38:2459–72. 10.1093/eurheartj/ehx144 28444290 PMC5837225

[45] Stone NJ Grundy SM. The 2018 AHA/ACC/Multi-Society Cholesterol guidelines: Looking at past, present and future. Prog Cardiovasc Dis. (2019) 62:375–83. 10.1016/j.pcad.2019.11.005 31733217

[46] Zhao M Wang M Zhang J Ye J Xu Y Wang Z. Advances in the relationship between coronavirus infection and cardiovascular diseases. Biomed Pharmacother. (2020) 127:110230. 10.1016/j.biopha.2020.110230 32428835 PMC7218375

